# Prevalence of smoking among undergraduate students in the Kingdom of Saudi Arabia: A meta-analysis

**DOI:** 10.18332/tid/190797

**Published:** 2025-03-06

**Authors:** Naif H. Alanazi

**Affiliations:** 1 Department of Public Health, College of Health Sciences, Saudi Electronic University, Riyadh, Saudi Arabia

**Keywords:** tobacco, shisha, hookah, students smoking, smoking prevalence

## Abstract

**INTRODUCTION:**

In recent years, tobacco smoking has become a major public health concern in the Kingdom of Saudi Arabia (KSA), particularly among adolescents and college students. Hence, the current study aimed to collect the available evidence of smoking prevalence in KSA over the past decade (2014–2023) among college students in KSA and to find its pooled prevalence.

**METHODS:**

This meta-analysis obtained relevant studies from PubMed, published during the period April 2014 to June 2023. All articles published in English in study venues within the Kingdom of Saudi Arabia on smoking prevalence/ epidemiology/factors among undergraduate and/or college-going students were included. Systematic review and review articles were excluded. MedCalc was used to calculate the pooled prevalence (95% CI).

**RESULTS:**

Among the 258 initial pool of articles, 34 fulfilled the eligibility criteria. Further screening revealed only 34 articles met the inclusion criteria for this meta-analysis. These studies’ minimum and maximum sample sizes were 208 and 3322, respectively. The lowest prevalence was 7.9%, and the highest was 59.57%. Hence, using the random effect model, the pooled prevalence of this study was 24.5% (95% CI: 21.013–28.09).

**CONCLUSIONS:**

The pooled prevalence of smoking is found to be 24.5%. Periodical educational seminars and related content must be arranged for college/university students to minimize the prevalence; hence, by reducing the smoking prevalence, morbidity and mortality can be minimized.

## INTRODUCTION

Smoking is defined as the inhalation of the smoke of burnt tobacco^1^. There are various types of smoking, including cigarettes, cigars, cigarillos, bidis, shisha, and kretek pipes or waterpipes, etc.^2-4^. Nowadays, smokeless tobacco is becoming fashionable and popular in many parts of the globe^2^. Hence, smoking has become one of the top and most common factors among young people for their poor health and high-risk factors for chronic diseases such as lung cancer and heart disease^5^.

Smoking is known to induce significant health problems; while it is widespread among young and college students, it can be controllable through preventive measures^6-8^. In 2019, it was reported that smoking has emerged as a significant issue of public health as it caused 8.71 million deaths and 229.77 DALYs (disability-adjusted life years) worldwide^9^. Among these deaths, 30% were due to smoking-induced cancer^7^.

It is suggested that college students face an elevated susceptibility to smoking, primarily attributed to a heightened probability of ready access to cigarettes^6,10^. Several modifiable factors exist to mitigate or regulate smoking prevalence among young students, with a notable factor being their academic tenure, particularly among final-year students who are believed to exhibit a greater inclination for smoking than their first-year counterparts. Additionally, the influence of family history, peer smoking behavior, and the broader social environment exerts a substantial impact on the initiation and continuation of smoking habits^11^.

The escalating prevalence of cigarette and tobacco smoking has emerged as a significant public health concern within the Kingdom of Saudi Arabia (KSA), particularly impacting adolescents and college students^12,13^. Recognizing the magnitude of this issue, identifying accumulated prevalence rates and pertinent influencing factors is crucial in formulating effective tobacco consumption prevention strategies^14^.

Hence, the current study aimed to collect the available evidence of smoking prevalence over the past decade (2014–2023) among college students in KSA and to find its pooled prevalence.

## METHODS

### Study design

This study applied a systematic review and meta-analysis research design.

### Reporting method

A systematic search was conducted on smoking prevalence among undergraduate students in PubMed from 2014 to June 2023. The standard guidelines as per Preferred Reporting Items for Systematic Review and Meta-Analysis Statement (PRISMA) were utilized to ensure that all relevant information was presented clearly and comprehensively.

### Search strategy and selection criteria

A systematic search was conducted for all English-language articles originating from the KSA on smoking prevalence, epidemiology, and associated factors among undergraduate, college-going, or secondary school students. The search process employed Boolean operators (AND/OR) and utilized keywords such as: ‘smoking among students’, ‘prevalence of smoking among students’, ‘cigarette smoking’, etc.

### Inclusion criteria

All free full-length articles published in selected search engines, with selected keywords, in the English language with clear objectives having prevalence/ frequency/behavior or status of smoking, were included.

### Exclusion criteria

**Figure 1:**
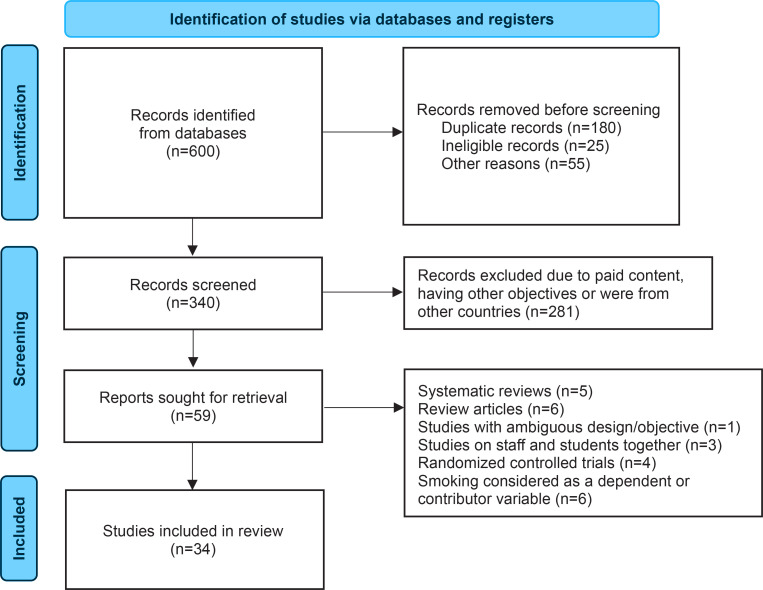
The study PRISMA flowchart

Previous systematic reviews and review articles^15-20^ and other articles were excluded if they were conducted on staff and students, any RCTs, where the impact of tobacco on any health-related issue was seen, and where smoking was considered a dependent/contributor variable. Figure 1 shows the study PRISMA flowchart.

### Data extraction technique

A data extraction form was designed to collect data from the short-listed studies. The author’s information, year, research design, sample size, prevalence of smoking, and study population were all included on the form. To facilitate a comprehensive reassessment of the articles and rectify any disparities or partialities in the presented data, a rigorous deliberation was undertaken with two experts in the respective field to reduce potential biases. The disagreement or ambiguity in the recovered data was resolved by having a conversation and coming to an agreement.

### Statistical analysis

Microsoft Excel was used to create a meta-analysis table that included the research headings: study, total numbers taken in the study, number of smokers, and its percentage (considered as prevalence). The application medCalc was used to calculate the pooled prevalence (95% CI). Funnel and forest plots were used. The heterogeneity test was conducted using Q statistics and I^2^, and publication bias was tested through Egger’s and Begg’s tests (Kendall’s tau); a p<0.05 was considered significant. Due to high I^2^ values – more than 75%, which is regarded as a strong heterogeneity – a random effects models was used^21,22^.

## RESULTS

Among the 258 initial pool of articles, only 34 articles were selected in this study and used for further meta-analysis (Figure 1). Most articles were conducted on medical students. The minimum and maximum sample sizes taken in these studies were 208^23^ and 3322^24^. The lowest prevalence was 7.9%^25^ (95% CI: 5.19–11.46) in male university students. The other lowest prevalence among medical students was 10.6%^26^ (95% CI: 7.07–14.97). One of the highest prevalence was reported as 50.5%^23^ (95% CI: 43.48–57.47) and 59.57%^27^ (95% CI: 54.38–64.60) among specifically male university students and overall university students. Table 1 shows the study characteristics and smoking pooled prevalence.

**Table 1:** Studies characteristics and pooled prevalence of smoking

*Authors Year*	*Total* *n*	*Smokers*			*Weight*		*Study population*
		*n*	*%*	*95% CI*	*Fixed*	*Random*	
Bin Abdulrahman et al.^28^ 2022	895	307	34.3	31.20–37.51	4.25	3.00	Medical students
Albangy et al.^29^ 2019	240	98	40.8	34.554–47.342	1.14	2.85	Male secondary school students
Almogbel et al.^30^ 2021	928	185	19.93	17.410–22.653	4.40	3.00	University students
Alkhalaf et al.^31^ 2021	354	44	12.4	9.178–16.325	1.68	2.91	Medical students
Alnasser et al.^32^ 2022	421	107	25.4	21.323–29.857	2.00	2.94	Medical students
Alwhaibi et al.^33^ 2022	675	137	20.3	17.323–23.532	3.21	2.98	Pharmacy students
Almogbel et al.^25^ 2020	316	25	7.9	5.185–11.457	1.50	2.90	Male university students
Ahmad et al.^34^ 2021	388	77	19.84	15.991–24.167	1.84	2.93	Medical students
Al-Qahtani^35^ 2022	454	117	25.77	21.807–30.054	2.16	2.94	University students
Algorinees et al.^12^ 2016	287	56	19.5	15.089–24.577	1.37	2.88	Medical students
Alenazi et al.^36^ 2023	400	111	27.8	23.416–32.417	1.90	2.93	Male high school students
Mansour^37^ 2017	336	84	25.0	20.460–29.987	1.60	2.91	Dental students
Qanash et al.^38^ 2019	1007	142	14.1	12.009–16.405	4.78	3.00	Health science students
Alshayban and Joseph^39^ 2019	464	106	22.8	19.101–26.939	2.20	2.95	University students
Salih et al.^40^ 2020	405	138	34.0	29.466–38.917	1.92	2.93	University students
Aqeeli et al.^41^ 2022	775	163	21.0	18.213–24.074	3.68	2.99	Undergraduate students
Almutairi^42^ 2016	715	213	29.8	26.457–33.291	3.39	2.98	Male college students
Almutairi^42^ 2016	492	112	22.7	19.131–26.729	2.34	2.95	Male college students
Sharanesha et al.^43^ 2022	400	176	44.0	39.071–49.019	1.90	2.93	Dental students
Almutairi^44^ 2014	1789	202	11.3	9.861–12.850	8.49	3.03	Medical students
Alzahrani et al.^23^ 2021	208	105	50.5	43.482–57.466	0.99	2.82	Male university students
Al-Zalabani^45^ 2015	870	181	20.8	18.152–23.656	4.13	3.00	Male secondary school students
Ansari et al.^46^ 2016	340	95	28.0	23.235–33.036	1.62	2.91	College students
Awan et al.^47^ 2016	535	198	37.0	32.906–41.257	2.54	2.96	Health science students
AlSwuailem et al.^48^ 2014	400	68	17.0	13.450–21.047	1.90	2.93	Dental students
Al-Zalabani and Kasim^24^ 2015	3322	504	15.2	13.968–16.437	15.75	3.04	Intermediate and secondary schools
Muzammil et al.^27^ 2019	371	221	59.6	54.380–64.603	1.76	2.92	University students
Ansari and Farooqi^49^ 2017	332	44	13.3	9.797–17.381	1.58	2.90	Female medical students
Habib et al.^50^ 2020	401	49	12.2	9.178–15.831	1.91	2.93	Medical students
Albgami et al.^51^ 2023	319	128	40.1	34.704–45.732	1.52	2.90	Male medical students
Rayes et al.^52^ 2023	534	109	20.6	17.071–24.084	2.54	2.96	Adolescent population
Almutham et al.^26^ 2019	256	27	10.6	7.066–14.973	1.22	2.86	Medical students
Alshanberi et al.^53^ 2021	910	289	31.8	28.742–34.893	4.32	3.00	Medical students
Alzahrani et al.^54^ 2023	519	125	24.0	20.466–28.001	2.47	2.96	Medical students
Total (fixed effects)	21058		21.9	21.317–22.438	100	100	
Total (random effects)	21058		24.5	21.013–28.090	100	100	

**Figure 2:**
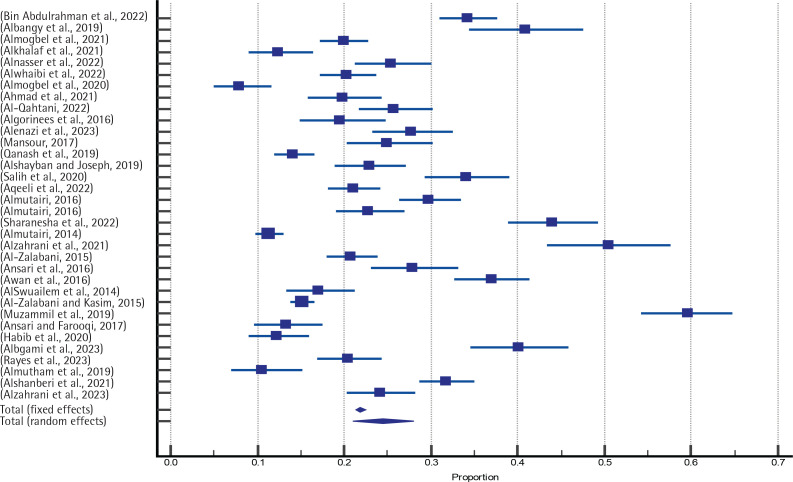
Forest plot showing the prevalence of smoking among college students in the Kingdom of Saudi Arabia from 2014 to 2023 in different studies (the error bars show the 95% confidence interval for each prevalence)

**Figure 3:**
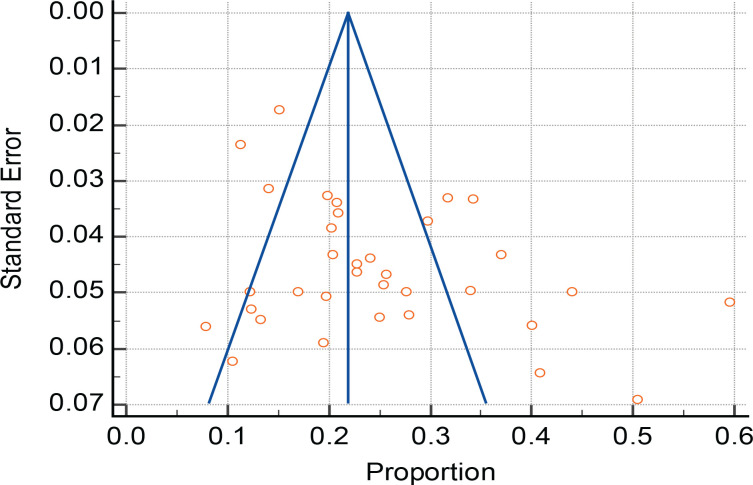
Funnel plot showing the publication bias

The test of heterogeneity was determined by Q-test (1188.026, p<0.0001) and I^2^ test [97.22% (95% CI: 96.70–97.66)]; both indicating a strong heterogeneity. Therefore, the random effects model was used for pooled prevalence. The publication bias was tested using Egger’s test [7.9934 (95% CI: 2.2482 –13.7385), p=0.0079] and Begg’s test/Kendall’s tau (p=0.2463), indicating that it was insignificant. A forest plot was applied to provide a visual indication of the degree of study heterogeneity and the estimated common impact. A funnel plot was utilized to compare the findings and precision of individual studies or the degree to which the estimated intervention effect size is near the genuine effect size. So, there was no relationship between effect size and its standard error (Figures 2 and 3). Hence, using the random effect model, the pooled prevalence was 24.6% (21.01–28.09) (Table 1).

## DISCUSSION

Tobacco consumption is still the world’s greatest health concern among college students^28^. Tobacco smoking among college students presents a pressing public health concern involving various physical, mental, and social challenges. It has become one of the leading factors or causes of illness, bad health, and death^28^. The college environment, usually characterized by academic stressors, feelings of independence, and peer influences, can contribute to the initiation and continuation of smoking habits^55^. The consequences of tobacco use during these formative years extend beyond immediate health risks, including long-term implications for both individuals and the broader community^55^. Smoking can adversely affect students’ academic performance, exacerbating stress and hindering concentration. Studies have shown that smoking causes an estimated 8 million premature deaths annually^55^.

This meta-analysis study indicated the pooled prevalence was 24.5% (95% CI: 21.013–28.090). Nevertheless, a meta-analysis conducted on Iranian students reported that the pooled prevalence of smoking was 19.8% (95% CI: 17.7–21.9) among male students and 2.2% (95% CI: 1.4–3.02) among female students^56^. Furthermore, two meta-analyses from Ethiopian university students reported pooled prevalence of the current status of smoking as 15.9% (95% CI: 12.21–19.63)^57^ and 12.55% (95% CI: 10.39–14.72), with no publication bias^58^. In the current meta-analysis, the pooled prevalence is high, signaling a notably elevated incidence compared to findings in other relevant studies. This higher prevalence underscores the significance of the observed phenomenon and suggests a potentially heightened public health concern compared to existing research.

It is well-known that the average onset of smoking occurs during the younger age^28^, which has made smoking a concerning issue among college students^18^. In one study, a response rate of 38.02% was achieved from a sample of 354 students, comprising 51.7% males and 48.3% females. Among them, 12.4% of medical students reported smoking occasionally, while 39.9% of all medical students reported they were exposed to passive smoking. According to the study, 5.9% of female and 18.6% of male medical students smoked regularly. With regard to the type of tobacco used, the study showed that 47% of smokers who were males did so using a waterpipe, compared to 77.8% of smokers who were females^31^.

Yet another study revealed that, of the 895 students who answered the poll, the majority (76.4%) said they had never tried or smoked tobacco. The majority of smokers started during the past five years (46.4%), suggesting that they probably started as soon as they enrolled in college. When they were asked whether they would like to smoke, the majority of students (57.1%) responded they would when they were anxious or under pressure. There was a significant correlation (53.1%) between smoking and having a family member who smokes^28^. In a study conducted on 421 respondents, 243 (57.7%) were aged 18–24 years, 255 (60.6%) were females, and 164 (39%) were from the Eastern Province of KSA. Males had a larger prevalence of smoking than females, with 44% and 13.3%, respectively (p<0.001), making the total prevalence of smoking 25.4%^32^.

Even though the prevalence of non-smokers among pharmacy students is high, there is a limited understanding of modifiable determinants contributing to the escalating trend. Therefore, it is advisable to promptly initiate awareness programs to guide students in abstaining from smoking, allowing them to concentrate on their studies while maintaining optimal physical and mental health^33^.

A study showed that among secondary school students, cigarette smoking was the most prevalent form of smoking (67.3%), followed by shisha smoking (22.4%). Only 2.1% of students reported smoking in other ways, such as hashish, etc. Of the individuals surveyed, 29.6% smoked more than five cigarettes a day, accounting for 39.8% of daily smokers^29^. Similarly, 336 dentistry students participated in another study; of them, 25% reported using tobacco products either currently or in the past, and 96% reported having exposure to passive smoke. Approximately 50% of smokers started during the study of the dentistry program^37^.

Studies show that students in KSA have insufficient knowledge about the hazards, addiction, and health consequences of smoking. In order to minimize the prevalence of smoking among college students, education and awareness programs must be given a high priority in the medical school curriculum^59^. Moreover, it is imperative to start tobacco control programs in order to limit and/or prevent tobacco smoking^30,37^. Furthermore, effective measures should be maintained, such as a combination of smoke-free legislation, higher tobacco prices, easier access to therapies for quitting smoking, and media campaigns against tobacco use^7^. In order to improve tobacco-related global health and decrease smoking among medical students, doctors, and patients, a medical curriculum on tobacco-related health concerns and smoking cessation should be made mandatory^60^.

### Limitations

The results of this systematic review and meta-analysis may not fairly represent the smoking prevalence among particular demographics or subgroups within the undergraduate student body, making them inapplicable to all Saudi Arabian undergraduate students.

## CONCLUSIONS

As the pooled prevalence of smoking is high, it is recommended to take immediate interventions to control smoking through educational programs targeted at college students to improve their awareness about tobacco smoking and its health hazards. Moreover, educational programs should be a continuous process of quality control and maintenance to ensure the minimization of smoking and subsequently associated morbidity and mortality with it.

## Data Availability

Data sharing is not applicable to this article as no new data were created.
